# The BREAST-Q Implant Surveillance Module (BREAST-Q IS) As a Predictor of Breast Implant Revisional Surgery

**DOI:** 10.1093/asj/sjaf128

**Published:** 2025-06-28

**Authors:** Michelle Merenda, Arul Earnest, Rasa Ruseckaite, Elisabeth Elder, Patrick Garduce, Susannah Ahern

## Abstract

**Background:**

The Australian Breast Device Registry (ABDR) records breast implant surgeries Australia-wide. In addition to clinical data, the ABDR has collected patient-reported outcome measures (PROMs) to better understand patient outcomes following implant surgery.

**Objectives:**

Our objective was to assess the association between postoperative PROMs and revisional surgery due to complications for reconstructive and cosmetic breast implant patients.

**Methods:**

A cohort study design was performed. All primary breast augmentation and breast reconstruction implant insertion procedures with 2 years of follow-up after PROM response, and with at least 1 PROM completed between October 30, 2017, and May 16, 2021, registered with the ABDR were identified. The primary outcome investigated was complications requiring revision at 2 years post PROM completion. Binary logistic regression models were applied to assess the predictive ability of PROMs.

**Results:**

A total of 5321 reconstructive procedures and 25,777 cosmetic breast procedures were followed. Multivariate regression for the reconstructive cohort showed that 3 PROM variables, feel, rippling, and tightness, predicted revision due to complications within 2 years of PROM response (OR 0.71, 95% CI 0.56-0.90, *P* = .004; OR 0.70, 95% CI 0.57-0.87, *P* < .001; and OR 0.80, 95% CI 0.69-0.93, *P* = .003, respectively). Multivariate regression for the cosmetic cohort showed that 3 PROM variables, look, rippling, and tightness, predicted revision due to complications within 2 years of PROM response (OR 0.51, CI 0.42-0.63, *P* < .001; OR 0.78, CI 0.63-0.95, *P* = .014; and OR 0.79, CI 0.67-0.94, *P* = .006, respectively).

**Conclusions:**

Postoperative PROMs were significantly associated with revisional surgery due to complications. PROMs may be employed to predict the likelihood of complications and revision following breast implant surgery.

**Level of Evidence: 3 (Therapeutic):**

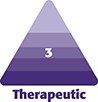

Breast implant surgery is the most common aesthetic surgical procedure performed by plastic surgeons worldwide.^1^ In addition, breast implants are utilized for breast reconstruction after breast cancer or for congenital defects, underscoring the gravity of their use. Breast implants, classed as high-risk medical devices, can pose a risk to implant recipients.^2,3^ Possible risks of implant insertion include shorter-term complications such as seroma or hematoma and deep wound infection, and middle to longer-term complications including malposition, capsular contracture, and skin scarring, all of which may lead to revisional surgery and its associated risks.^4,5^ Most complications from breast implant surgery are associated with the patient experiencing breast pain, rippling, or tightness, prompting the individual to seek clinical review or care. However, although symptoms may prompt individuals to seek clinical care, many do not for a variety of reasons, such as low awareness, perceived low severity, dissatisfaction with the decision-making process, depression or anxiety, lack of continuity of care in patients who do not have a regular provider, or normalization of symptoms. Access barriers include cost concerns, geographic limitations, lack of specialized services, and long wait times. Together, these factors contribute to delays in diagnosis and treatment, potentially complicating outcomes.^6-8^

Patient-reported outcome measures (PROMs) are questionnaires that aim to provide information from the patient regarding their recovery following treatment, or regarding their general well-being.^9^ Understanding patient outcomes and concerns regarding implants through the collection of PROMs following surgery is an additional source of information regarding surgical and device outcomes that can complement clinical measures because they may provide useful information regarding the patient's clinical outcome that the surgeon may not otherwise be aware of, and that may be clinically relevant.^9^

A recent scoping review found that, for high-risk medical device registries, the use of PROMs as a tool to predict potential revisional surgery due to complications has been underutilized.^10^ Studies reporting on PROMs in high-risk medical device registries are predominantly concerned with PROM data as an outcome measure, with only a small number of studies utilizing the PROM as a predictor of future outcomes itself.^11-15^ Moreover, there were no predictive studies of PROM data from breast device registries. Studies of high-risk medical device registries that evaluated PROMs as a predictor undertook regression analysis and built PROMs into the statistical model as a risk factor.^11-15^ Studies considering PROMs in high-risk medical device registries, such as the New Zealand Joint Registry, an orthopedic registry that monitors joint replacement devices (knee, hip, etc); the Interagency Registry for Mechanically Assisted Circulatory Support (INTERMACS), a cardiac registry that monitors cardiac implantable electronic devices such as continuous-flow left ventricular assist devices; and the Danish Shoulder Arthroplasty Register, are examples of registries that have employed PROMs as predictors of future outcomes.^11-15^ These studies found that PROMs collected at earlier time points after surgery were associated with PROMs or revision outcomes at later time points, and therefore could potentially identify individuals at risk of complications or return to surgery. Identifying breast implant patients at risk of early revision potentially enables the surgeon to monitor their patient's progress more frequently and intervene promptly.

The Australian Breast Device Registry (ABDR) is a commonwealth-funded registry that was established to record breast implant surgeries throughout Australia. The purpose of the registry is to report on possible trends and complications associated with breast device (implants, tissue expanders, and matrix/mesh) surgery; to monitor the long-term safety of breast implants; and to identify best surgical practice and optimal patient health outcomes.^16^ In addition to clinical outcomes, the ABDR has collected PROMs at 1, 2, and 5 years following primary implant insertion.

To our knowledge, this is the first time a breast device registry has been studied to evaluate whether PROMs may be associated with revision due to complications following breast implant surgery. This study presents a novel approach to the analysis of PROM data in a breast implant registry and provides a unique opportunity for establishing PROMs as a tool for predicting revisional surgery due to complications in women with breast implants with the aim of improving outcomes.

We analyzed data from the BREAST-Q implant surveillance module (BREAST-Q IS) collected by the ABDR after breast implant surgery to determine if there was a relationship between PROM outcomes and revision due to complications following breast implant insertion. The objective of this study was to establish the ability of the BREAST-Q IS to predict revisional surgery due to complications within 2 years of the first completed PROM response and to allow direct estimation of risk factors with data collected by the ABDR.

## METHODS

### Study Design and Setting

This was a retrospective cohort study employing the 5-question BREAST-Q IS. The BREAST-Q IS was developed specifically for a registry setting to discern adverse events following surgery.^17,18^ PROM variables include pain, tightness of the breast area, rippling, look, and feel. Responses pertaining to pain and tightness include “none of the time,” “a little of the time,” “some of the time,” “most of the time,” and “all of the time.” Response options for rippling, look, and feel questions are “very dissatisfied,” “somewhat dissatisfied,” “somewhat satisfied,” and “very satisfied.” PROMs are sent by short message service (SMS) to all eligible registry participants at 1, 2, and 5 years after primary implant insertion. Each SMS contains a unique link for the participant to complete the PROM online. When there is no mobile phone number, an email containing a unique link is sent in lieu of an SMS. If neither a mobile number nor email is available, the patient is sent a postal PROM. If a participant does not complete the PROM, they receive a follow-up phone call 14 days after the initial SMS, followed by 2 additional calls at 7-day intervals if needed. PROMs were collected from October 30, 2017, to May 16, 2021.

### Study Population

The ABDR is a national clinical quality registry that has collected approximately 30,000 PROMs from women who participate. The registry records a capture rate of 71% to 76% from Australian surgeons that perform breast device surgery.^16^ All primary breast augmentation and breast reconstruction implant insertion procedures with at least 2 years of postoperative follow-up and with at least 1 BREAST-Q IS questionnaire completed between October 30, 2017, and May 16, 2021, were identified in the ABDR. An implant was classified based on the available history of the breast in which it was inserted. Primary implants were defined as those which were inserted into breasts that had no in-situ breast implant (ie, procedure was not a replacement of an implant) and also had no recorded history of previous procedures involving implants recorded in the registry. The remaining implants inserted were classified as legacy implants.^16^ The PROM was administered at 1, 2, or 5 years after permanent implant insertion. This study considered only primary implants and any subsequent revisions of these. Revision could occur any time after implant insertion. All patient PROM data collected by the ABDR from all participating sites were eligible for inclusion.

### Inclusion and Exclusion Criteria

The primary outcome investigated was revision due to any of the ABDR's standardized list of complications within 2 years of PROM response, excluding patient preference. Patients who had any revisional surgery before their first PROM follow-up were excluded. Patients who did not complete all 5 PROM questions were excluded. Registered primary breast device surgery that did not have any follow-up PROM was also excluded. For patients who completed more than 1 PROM, the earliest PROM was taken; this was a valid method as shown by the sensitivity analysis. Revision due to patient preference was defined as revision without medical complications, for example, increase or decrease in implant size, or other patient preference factors including explantation. Revision due to complications cases were defined as patients whose initial surgery had been registered with the ABDR and who had gone on to have revisional surgery due to specific complications (eg, device malposition, capsular contraction, anaplastic large cell lymphoma, seroma or hematoma, device deflation, skin scarring problems, deep wound infection, breast cancer).

The primary breast device surgeries were divided into 2 cohorts, breast reconstruction or cosmetic.

### Ethics and Consent

The ABDR has an opt-out approach to patient consent. Participants are recruited to the registry by their clinician. To date, the ABDR has an opt-out rate of less than 1%, indicating extremely strong support from the eligible patient cohort. The ABDR has ethics approval from the Alfred Health and Monash Health human research ethics committees (HRECs), and this study received ethics approval from the Alfred Health Human Research Ethics Committee Project 742/20, Melbourne, Australia.

### Variables

The preoperative indication for all primary breast device surgeries (either reconstructive or cosmetic) was recorded prospectively at the time of surgery. Furthermore, covariates for all primary breast device surgeries were collected at the time of surgery. Revisional surgery and complications were recorded for those procedures that resulted in an operative procedure in the operating room.

### Sample Size

A sample size calculation was not performed because the study employed all available registry data.

### Statistical Analyses

Data analysis was stratified by reconstruction vs cosmetic because these were deemed a priori to be very different cohorts. Data were analyzed at breast level. Summary statistics described the characteristics of the cohort. PROM responses were treated as continuous to predict revision due to complications because they had the same prediction ability as keeping original categorical PROM responses while being simpler, and avoided information loss that might happen when collapsing categories. We completed a sensitivity check to test whether accounting for time elapsed from breast implant insertion to PROM completion date affected the results, that is, time from insertion to PROM, to account for the variation in PROM time points, for example, first available PROM, and first-year vs second-year or fifth-year PROM.

We performed univariate and multivariate logistic regression analysis to investigate the relationship between postoperative PROMs and risk of revision. Separate univariate analysis was also undertaken for each PROM question and specific complications at revision. Multivariate models for predicting specific complications were not considered due to concerns regarding small volumes of cases and lack of power. For the relationship between postoperative PROMs and risk of revision due to (any) complication, separate multivariate logistic regression models were completed for the 2 cohorts, with results expressed as adjusted odds ratios (ORs) with 95% confidence intervals (CIs). Independent variables were considered for inclusion in the logistic regression model if the *P* value was < .05 in univariate analysis. Forward stepwise regression was performed to determine variables for inclusion in each multivariate model. Starting with the variable most strongly associated with complications (within 2 years of PROM response), according to the univariate analysis, the Wald test (with a threshold *P* value of .05) was performed to examine whether the inclusion of the next most significant factor sufficiently improved the fit of the multivariate model. This was conducted sequentially until all variables were evaluated. Receiver operating characteristic (ROC) analysis was undertaken for the final model for each cohort to assess performance.

The sensitivity, specificity, negative predictive value, positive predictive value, and accuracy were calculated for each cohort based on the optimal final prognostic score. A minimally acceptable sensitivity of 70% was selected for the BREAST-Q IS detecting revisions due to complications within the following 2 years based on clinical relevance. Data analysis was undertaken in Stata V17 (Stata Corp., College Station, TX), and level of significance was set at 5%.^19^

## RESULTS

**Figure 1.**
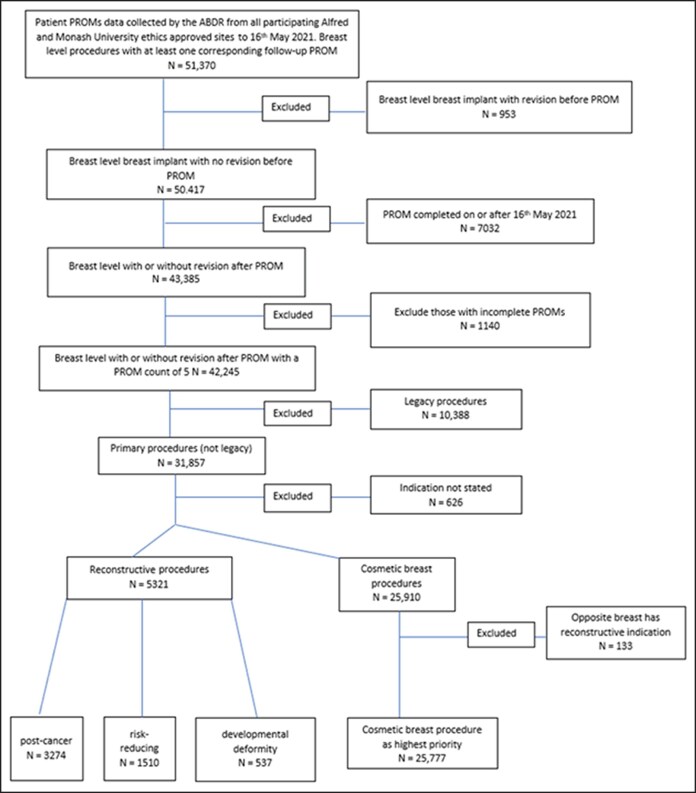
Australian Breast Device Registry patient-reported outcome measure data, breast level study flowchart.

Of the 51,370 breast level procedures with corresponding PROM data, 20,272 were excluded (Figure 1). There were 5321 reconstructive procedures and 25,777 cosmetic breast procedures with PROMs that were followed up for 2 years. All participants were female.

Baseline characteristics of the reconstructive cohort are shown in Table 1. Age range for the reconstructive cohort was 15.8 to 81.9 years. Overall mean age for the reconstructive cohort was 46.8 years (SD 12.5). In the reconstructive cohort, the age group 40-49 experienced a higher number of complications (42.1% of total complications). This was followed by age group 50-59 (26.4%). The overall revision rate for the reconstructive cohort was 3.7% (197 of 5321).

**Table 1.** Demographic and Clinical Characteristics of Reconstructive Patients

	No complications within 2 years	Complications (any) within 2 years	Total	*P* value
	*n* = 5124	*n* = 197	*n* = 5321	
Age				<.001
<30	538 (10.5%)	6 (3.0%)	544 (10.2%)	
30-39	923 (18.0%)	28 (14.2%)	951 (17.9%)	
40-49	1562 (30.5%)	83 (42.1%)	1645 (30.9%)	
50-59	1346 (26.3%)	52 (26.4%)	1398 (26.3%)	
≥60	755 (14.7%)	28 (14.2%)	783 (14.7%)	
Site type				.78
Public hospital	1076 (21.0%)	43 (21.8%)	1119 (21.0%)	
Private facility	4048 (79.0%)	154 (78.2%)	4202 (79.0%)	
Operation year				.031
2013	38 (0.7%)	5 (2.5%)	43 (0.8%)	
2014	192 (3.7%)	7 (3.6%)	199 (3.7%)	
2015	253 (4.9%)	6 (3.0%)	259 (4.9%)	
2016	481 (9.4%)	21 (10.7%)	502 (9.4%)	
2017	1281 (25.0%)	60 (30.5%)	1341 (25.2%)	
2018	1628 (31.8%)	61 (31.0%)	1689 (31.7%)	
2019	1197 (23.4%)	37 (18.8%)	1234 (23.2%)	
2020	54 (1.1%)	0 (0.0%)	54 (1.0%)	
Previous radiation therapy				.86
No	4034 (78.7%)	157 (79.7%)	4191 (78.8%)	
Yes	614 (12.0%)	24 (12.2%)	638 (12.0%)	
Not stated	476 (9.3%)	16(8.1%)	492(9.2%)	
Acellular dermal matrix/mesh use				.005
No	3859 (75.3%)	151 (76.6%)	4010 (75.4%)	
Yes	973 (19.0%)	45 (22.8%)	1018 (19.1%)	
Not stated	292 (5.7%)	1 (0.5%)	293 (5.5%)	
Surgical plane				.43
Subglandular/subfascial	437 (8.5%)	12 (6.1%)	449 (8.4%)	
Subpectoral/dual plane	3253 (63.5%)	119 (60.4%)	3372 (63.4%)	
Subflap	448 (8.7%)	22 (11.2%)	470 (8.8%)	
Other	133 (2.6%)	6 (3.0%)	139 (2.6%)	
Not stated	853 (16.6%)	38 (19.3%)	891 (16.7%)	
Patient level procedure type				.82
Unilateral	1127 (22.0%)	42 (21.3%)	1169 (22.0%)	
Bilateral	3997 (78.0%)	155 (78.7%)	4152 (78.0%)	
Nipple sparing				.026
No	3770 (73.6%)	133 (67.5%)	3903 (73.4%)	
Yes	1223 (23.9%)	62 (31.5%)	1285 (24.1%)	
Not Stated	131 (2.6%)	2 (1.0%)	133 (2.5%)	
Operation indication				.008
Post cancer	3142 (61.3%)	132 (67.0%)	3274 (61.5%)	
Risk reducing	1452 (28.3%)	58 (29.4%)	1510 (28.4%)	
Developmental deformity	530 (10.3%)	7 (3.6%)	537 (10.1%)	
Operation type				.77
Direct to implant	2234 (43.6%)	88 (44.7%)	2322 (43.6%)	
2-stage implant	2890 (56.4%)	109 (55.3%)	2999 (56.4%)	
PROM				
Look				<.001
Very dissatisfied	383 (7.5%)	38 (19.3%)	421 (7.9%)	
Somewhat dissatisfied	918 (17.9%)	71 (36.0%)	989 (18.6%)	
Somewhat satisfied	2199 (42.9%)	54 (27.4%)	2253 (42.3%)	
Very satisfied	1624 (31.7%)	34 (17.3%)	1658 (31.2%)	
Feel				<.001
Very dissatisfied	294 (5.7%)	39 (19.8%)	333 (6.3%)	
Somewhat dissatisfied	853 (16.6%)	49 (24.9%)	902 (17.0%)	
Somewhat satisfied	2289 (44.7%)	86 (43.7%)	2375 (44.6%)	
Very satisfied	1688 (32.9%)	23 (11.7%)	1711 (32.2%)	
Rippling				<.001
Very dissatisfied	307 (6.0%)	35 (17.8%)	342 (6.4%)	
Somewhat dissatisfied	874 (17.1%)	70 (35.5%)	944 (17.7%)	
Somewhat satisfied	1480 (28.9%)	32 (16.2%)	1512 (28.4%)	
Very satisfied	2643 (48.1%)	60 (30.5%)	2523 (47.4%)	
Pain				<.001
All of the time	67 (1.3%)	12 (6.1%)	79 (1.5%)	
Most of the time	230 (4.5%)	31 (15.7%)	261 (4.9%)	
Some of the time	845 (16.5%)	35 (17.8%)	880 (16.5%)	
A little of the time	1410 (27.5%)	50 (25.4%)	1460 (27.4%)	
None of the time	2572 (50.2%)	69 (35.0%)	2641 (49.6%)	
Tightness				<.001
All of the time	285 (5.6%)	30 (15.2%)	315 (5.9%)	
Most of the time	511 (10.0%)	34 (17.3%)	545 (10.2%)	
Some of the time	782 (15.3%)	44 (22.3%)	826 (15.5%)	
A little of the time	1219 (23.8%)	39 (19.8%)	1258 (23.6%)	
None of the time	2327 (45.4%)	50 (25.4%)	2377 (44.7%)	
Time between implant insertion and PROM response				.93
1 year	3362 (65.6%)	130 (66.0%)	3492 (65.6%)	
2 years	1278 (24.9%)	50 (25.4%)	1328 (25.0%)	
5 years	484 (9.4%)	17 (8.6%)	501 (9.4%)	

PROM, patient-reported outcome measure.

Baseline characteristics of the cosmetic cohort are shown in Table 2. Age range for the cosmetic cohort was 16.4 to 85.7 years. Overall mean age for the cosmetic cohort was 33.4 years (SD 9.5).The age group with the highest number of complications in the cosmetic cohort was <30 (44.6% of total complications), followed by age group 30-39 (28.9%). The overall revision rate for the cosmetic cohort was <1% (249 out of 25,777).

**Table 2.** Demographic and Clinical Characteristics of Cosmetic Patients

	No complications within 2 years	Complications (any) within 2 years	Total	*P* value
	*n* = 25528	*n* = 249	*n* = 25777	
Age				.031
<30	10346 (40.5%)	111 (44.6%)	10457 (40.6%)	
30-39	9494 (37.2%)	72 (28.9%)	9566 (37.1%)	
40-49	4170 (16.3%)	51 (20.5%)	4221 (16.4%)	
50-59	1306 (5.1%)	15 (6.0%)	1321 (5.1%)	
≥60	212 (0.8%)	0 (0.0%)	212 (0.8%)	
Operation year				<.001
2013	66 (0.3%)	2 (0.8%)	68 (0.3%)	
2014	148 (0.6%)	7 (2.8%)	155 (0.6%)	
2015	939 (3.7%)	19 (7.6%)	958 (3.7%)	
2016	3316 (13.0%)	26 (10.4%)	3342 (13.0%)	
2017	8694 (34.1%)	97 (39.0%)	8791 (34.1%)	
2018	8048 (31.5%)	62 (24.9%)	8110 (31.5%)	
2019	4011 (15.7%)	36 (14.5%)	4047 (15.7%)	
2020	306 (1.2%)	0 (0.0%)	306 (1.2%)	
Surgical plane				.005
Subglandular/subfascial	2817 (11.0%)	40 (16.1%)	2857 (11.1%)	
Subpectoral/dual plane	21125 (82.8%)	200 (80.3%)	21325 (82.7%)	
Other	64 (0.3%)	2 (0.8%)	66 (0.3%)	
Not stated	1522 (6.0%)	7 (2.8%)	1529 (5.9%)	
Patient level procedure type	0.63			
Unilateral	24 (0.1%)	0 (0.0%)	24 (0.1%)	
Bilateral	25504 (99.9%)	249 (100.0%)	25753 (99.9%)	
PROM				
Look				<.001
Very dissatisfied	661 (2.6%)	42 (16.9%)	703 (2.7%)	
Somewhat dissatisfied	1805 (7.1%)	51 (20.5%)	1856 (7.2%)	
Somewhat satisfied	7604 (29.8%)	79 (31.7%)	7683 (29.8%)	
Very satisfied	15458 (60.6%)	77 (30.9%)	15535 (60.3%)	
Feel				<.001
Very dissatisfied	446 (1.7%)	22 (8.8%)	468 (1.8%)	
Somewhat dissatisfied	1543 (6.0%)	35 (14.1%)	1578 (6.1%)	
Somewhat satisfied	7166 (28.1%)	99 (39.8%)	7265 (28.2%)	
Very satisfied	16373 (64.1%)	93 (37.3%)	16466 (63.9%)	
Rippling				<.001
Very dissatisfied	521 (2.0%)	28 (11.2%)	549 (2.1%)	
Somewhat dissatisfied	1565 (6.1%)	38 (15.3%)	1603 (6.2%)	
Somewhat satisfied	3643 (14.3%)	43 (17.3%)	3686 (14.3%)	
Very satisfied	19799 (77.6%)	140 (56.2%)	19939 (77.4%)	
Pain				<.001
All of the time	173 (0.7%)	10 (4.0%)	183 (0.7%)	
Most of the time	550 (2.2%)	8 (3.2%)	558 (2.2%)	
Some of the time	2435 (9.5%)	56 (22.5%)	2491 (9.7%)	
A little of the time	6352 (24.9%)	70 (28.1%)	6422 (24.9%)	
None of the time	16018 (62.7%)	105 (42.2%)	16123 (62.5%)	
Tightness				<.001
All of the time	154 (0.6%)	12 (4.8%)	166 (0.6%)	
Most of the time	388 (1.5%)	7 (2.8%)	395 (1.5%)	
Some of the time	1297 (5.1%)	34 (13.7%)	1331 (5.2%)	
A little of the time	3713 (14.5%)	44 (17.7%)	3757 (14.6%)	
None of the time	19976 (78.3%)	152 (61.0%)	20128 (78.1%)	
Time between implant insertion and PROM response				<.001
1 year	16199 (63.5%)	152 (61.0%)	16351 (63.4%)	
2 years	8085 (31.7%)	67 (26.9%)	8152 (31.6%)	
5 years	1244 (4.9%)	30 (12.0%)	1274 (4.9%)	

PROM, patient-reported outcome measure.

Refer to Table 3 for counts of complication type related to revisional surgery.

**Table 3.** Count of Complication Type Related to Revisional Surgery

Complication	Reconstruction (*n*)	Cosmetic (*n*)
Device malposition	94	107
Capsular contracture	97	115
Device rupture/deflation	14	37
Skin scarring	19	19
Seroma/hematoma	6	4
Deep wound infection	8	1

Univariate analysis for the reconstruction cohort showed that those <30 years of age were at lower risk of complications than those >40 years. It also revealed that all 5 PROM questions showed a significant association with future PROMs and revision due to complications within 2 years of the first completed PROM response for the breast reconstruction cohort. For each 1 point increase in PROM there was a reduction in odds ratio: look OR 0.54, 95% CI 0.44-0.65, *P* < .001; feel OR 0.50, 95% CI 0.42-0.61, *P* < .001; rippling OR 0.56, 95% CI 0.46-0.67, *P* < .001; pain OR 0.63, 95% CI 0.53-0.75, *P* < .001; and tightness OR 0.67, 95% CI 0.59-0.76, *P* < .001. For the cosmetic cohort all 5 PROM questions showed a significant association with PROMs and revision due to complications within 2 years of the first completed PROM response for the cosmetic cohort. For each 1 point increase in PROM there was a reduction in odds ratio: look OR 0.42, 95% CI 0.36-0.50, *P* < .001; feel OR 0.49, 95% CI 0.41-0.58, *P* < .001; rippling OR 0.52, 95% CI 0.44-0.62, *P* < .001; pain OR 0.60, 95% CI 0.52-0.70, *P* < .001; and tightness OR 0.60, 95% CI 0.51-0.70, *P* < .001.

Multivariate regression models for the reconstructive cohort showed that 3 PROM variables, feel, rippling, and tightness, predicted the outcome of revision due to complications excluding patient preference within 2 years of the first completed PROM response in this cohort: OR 0.71, 95% CI 0.56-0.90, *P* = .004; OR 0.70, 95% CI 0.57-0.87, *P* = .001; and OR 0.80, 95% CI 0.69-0.93, *P* = .003, respectively (Table 4). ROC analysis from logistic regression for PROMs and breast reconstruction area under the curve (AUC) value was 0.7134 (see Supplemental Figure 1, located online at https://doi.org/10.1093/asj/sjaf128).

**Table 4.** Multivariate Model for the BREAST-Q IS Individual Questions at 2-Year Follow-up for Primary Reconstructive Implants

	OR (95% CI)	*P* value
PROM feel	0.71 (0.56-0.90)	.004
PROM rippling	0.70 (0.57-0.87)	.001
PROM tightness	0.80 (0.69-0.93)	.003

BREAST-Q IS, BREAST-Q implant surveillance module; CI, confidence interval; OR, odds ratio; PROM, patient-reported outcome measure.

Multivariate regression models for the cosmetic cohort showed that 3 PROM variables predicted the outcome of revision due to complications excluding patient preference within 2 years of the first completed PROM response for this cohort: look, OR 0.51, 95% CI 0.42-0.63, *P* < .001; rippling OR 0.78, 95% CI 0.63-0.95, *P* = .014; and tightness, OR 0.79, 95% CI 0.67-0.94, *P* = .006 (Table 5). ROC analysis from logistic regression for PROMs and the cosmetic cohort AUC was 0.7179 (see Supplemental Figure 2, located online at https://doi.org/10.1093/asj/sjaf128). Supplemental Table 1 (located online at https://doi.org/10.1093/asj/sjaf128) shows univariate analysis for specific complications at revision vs each PROM for both cohorts.

**Table 5.** Multivariate Model for the BREAST-Q IS Individual Questions at 2-Year Follow-up for Cosmetic Implants

	OR (95% CI)	*P* value
PROM look	0.51 (0.42-0.63)	<.001
PROM rippling	0.78 (0.63-0.95)	.014
PROM tightness	0.79 (0.67-0.94)	.006

BREAST-Q IS, BREAST-Q implant surveillance module; CI, confidence interval; OR, odds ratio; PROM, patient-reported outcome measure.

## DISCUSSION

To our knowledge this is the first registry-based study to examine the ability of a PROM, in this case the BREAST-Q IS, to predict revisional surgery due to complications following breast implant insertion. In this study we found that the BREAST-Q IS a predictor of revision due to complications within 2 years of the first completed PROM response. Furthermore, BREAST-Q IS factors, including rippling and tightness, were common across both cohorts in discriminating revision due to complications. Multivariate analysis revealed that in the reconstruction cohort the 3 most significant PROM factors indicative of complications were feel, rippling, and tightness. In the cosmetic cohort, the 3 most significant PROM factors associated with complications leading to revision were look, rippling, and tightness. These findings support the available literature that PROMs can be utilized as a predictor of revisional surgery/complications in high-risk medical device registries.^11-15^

The overall revision rates between the 2 cohorts were different. This may be attributed to the very different nature of both procedures. The main difference is that a reconstruction after breast cancer, or in the case of risk-reducing (noncancer) reconstructions, occurs after a mastectomy when the breast tissue is removed, as opposed to a cosmetic procedure that occurs when the native breast is intact (or slightly modified in the case of a simultaneous mastopexy). Other treatments for cancer such as radiotherapy and chemotherapy also impact the complication rate. The complication profile and the outcome are different. The situation of reconstruction for developmental reasons is again a totally different situation due to congenital variations.

Rippling and tightness were predictive of revision due to complications in both cohorts. Rippling and tightness are multifactorial and reflect both patient-related and device-related factors. Rippling is commonly attributed to inadequate soft tissue coverage, particularly in individuals with a lean body mass, and may be exacerbated by factors such as implant type (eg, saline vs silicone), underfilling, or subglandular placement.^20,21^ Tissue atrophy over time and implant edge visibility further contribute to this phenomenon. Tightness is frequently reported in association with capsular contracture, although patients may describe a sensation of tightness even in the absence of overt clinical contracture.^22^ This may represent early capsular changes or fibrosis along scar planes. Although both symptoms are typically considered indicators of longer-term complications, our findings suggest they may also act as early markers of dissatisfaction or functional compromise, potentially prompting revisional surgery. Currently, the registry does not capture detailed data on patient-specific factors such as BMI or soft tissue thickness, which limits our ability to further delineate risk profiles for these symptoms.

A poor PROM response, that is, a response of “very dissatisfied’ in response to “feel” was significant in predicting revision due to complications in the reconstruction cohort but not for the cosmetic cohort and may suggest that reconstructive patients place greater importance on how the implant feels in situ than on a desired look. Look was significant in predicting revision due to complications in the cosmetic cohort but not for the reconstruction cohort. Unless the indication for breast implant surgery is to repair a congenital deformity, it would be fair to infer that the motivation behind breast implant surgery for the cosmetic cohort is primarily an aesthetic one, and therefore look is an outcome priority for cosmetic patients.

Interestingly, pain was not predictive of revisional surgery due to complications for either cohort in the multivariate models. This finding is surprising because it could be reasonably expected that pain would be an indicator of a complication requiring revision. In orthopedic studies by Hosman, and Rothwell, upon investigating PROMs and risk of revision it was found that pain was not ranked highly as an indication for revision.^9,11^ In contrast, a study by Gupta reported that pain was the most common reason for revision.^15^ The lack of predictive value of pain in our analysis may stem from several factors. Pain is a subjective and multifaceted experience influenced by individual thresholds, psychological state, and contextual factors, which can introduce considerable variability in self-reported measures. Additionally, pain may not be directly linked to the primary outcome of interest or may act as a mediator rather than an independent predictor, thereby weakening its statistical association in the model. Breast devices are a unique clinical situation in that a breast implant may not be considered functional the way a hip or knee implant is. This may account for a greater importance being placed on pain when a functional device is implanted. Pain experienced by a recipient of a functional implant may persist and affect quality of life, requiring immediate intervention, more so than it would if it were a breast implant, making surveillance a more appropriate response.

Our findings are supported by the existing literature, which show that PROMs can be optimized to predict adverse events.^11-15^ All 5 studies established the association between PROMs and the ability to predict adverse events such as revision, device failure, mortality, or pain through the use of regression analysis. Although there are no similar studies analyzing the predicative ability of the BREAST-Q IS in other breast device registries, the statistical methods adopted by other high-risk medical device registries can be extrapolated to a breast registry context. In a breast registry setting, this type of regression analysis for PROMs remains novel and should be explored as it has been by other registry types.

We explored the different ways to investigate the predictive ability of the BREAST-Q IS (see Supplemental Tables 2, 3, located online at https://doi.org/10.1093/asj/sjaf128). Similar to other non–breast device registries we looked at PROMs as predictors of adverse events through regression analysis, and built the 5 BREAST-Q IS questions into the statistical models as a risk factor. Justification for why PROM questions were treated as continuous variables can be seen in Supplemental Tables 2, 3. Consequently, a better PROM response translates to a better outcome; a poorer PROM response to a poorer outcome.

After completing the sensitivity analysis to account for utilizing the first available PROM, the odds ratios of the PROMs changed very little based on follow-up time point. There was no significant change in the magnitude of the odds ratio in the reconstructive or the cosmetic model. For the reconstruction multivariate model, feel, rippling, and tightness were statistically significant. For the cosmetic cohort look, rippling, and tightness were statistically significant. An ROC curve analysis indicated good discrimination ability for both models. We demonstrated that poor PROM responses are likely to be associated with an increased chance of revision due to complications.

### Implications

PROM outcomes from breast implantation evaluated with the Breast Q IS tool were shown in this study to be strongly associated with the need for revisional surgery due to complications. Although this study was the first of its type for breast devices, similar studies for other implanted medical devices have shown equivalent results. The administration of condition-specific PROMs postoperatively may therefore be an effective adjunct to routine clinical practice, by identifying individual postsurgical patients for potential early review. Although a number of national registries do regularly administer PROMs, it is acknowledged that this is not always the case. Nevertheless, PROM programs for clinical care may be feasible for larger group practices or hospital settings, and may warrant cost-benefit analysis at a local level.

We found that the utility of the BREAST-Q IS as a predictive tool provides a promising method for identifying complications that may lead to revisional surgery in the care of patients both before and after breast implant surgery and may be helpful in decision-making by clinicians. From a registry perspective, PROMS are an objective way of picking up symptoms earlier than data on revision. The finding of rippling and tightness as predictors of complications leading to revision provides the opportunity for surgeons to reassess the postsurgical management of patients presenting with rippling or tightness, and perhaps adjust surgical techniques to minimize such outcomes by mitigating the circumstances under which they occur. Postoperatively, surgeons can pay closer attention to a patient presenting with rippling or tightness, monitoring them more frequently, or even returning to surgery before symptoms are exacerbated. Conversely, patients with better responses are unlikely to require revision and could be reviewed less frequently. At a minimum, these findings may help facilitate conversations between surgeon and patient, particularly in the area of managing expectations around rippling and tightness.

### Limitations

Our study does have limitations. In our study we excluded complication revisions before PROMS to address the issue of reverse causality; however, we acknowledge the possibility that some patients may experience issues reflected in low PROM scores but may not proceed to revisional surgery, either due to personal choice, delays in access to care, or a decision to manage conservatively. As a result, although PROMs may reflect underlying complications, not all such cases may culminate in surgical intervention. This potential mismatch between symptom reporting and revision could lead to an underestimation of the true predictive value of PROMs. Another potential limitation of this study is selection bias, because patients who choose to complete PROMs may differ systematically from those who do not. It is often assumed that individuals experiencing complications or dissatisfaction are more likely to respond, potentially skewing results toward poorer outcomes among PROM responders. However, in our cohort, we found the opposite, because PROM responders were significantly less likely to undergo revisional surgery than nonresponders. This finding suggests that responders may represent a more engaged or healthier subset of patients, possibly due to higher health literacy, greater satisfaction, or stronger adherence to follow-up care. If selection bias is present, it may bias the association between PROMs and revision toward the null, underestimating rather than overstating the predictive utility of PROMs.

One noteworthy limitation of this study is that we opted to exclude revisions before PROMs to demonstrate a cause-effect relationship at the expense of underestimating the overall incidence of revisions. By excluding patients who had undergone revisional surgery before completing the PROMs we were able to investigate the ability of PROMs to predict revisions due to complication. What is more, the ABDR collects PROMs at 1, 2, and 5 years of follow-up, and the lack of a PROM survey at baseline or shortly after baseline limits the ability to predict revisions due to short-term complications. We do, however, acknowledge that this exclusion may result in an underreporting of short-term complications such as hematoma and infection, which typically occur within the first year postsurgery, and, as the reviewer has pointed out, potentially bias the study against capturing the full spectrum of complications leading to revisional surgery.

A univariate analysis of PROMs with complication type demonstrated that certain PROMs are more strongly associated with specific complications, particularly those related to device malposition, capsular contracture, and skin scarring, in both the reconstructive and cosmetic cohorts. Notably, look, feel, rippling, and tightness were the most consistently predictive across a range of complications. In contrast, complications such as seroma or hematoma and deep wound infection exhibited weaker associations with PROMs, possibly due to the lower frequency of these events. As the counts were low, we chose to pool all data together to increase the sample size and improve the reliability of the analysis. PROMs in the registry are scheduled based on fixed anniversaries and are not specifically designed to capture before-and-after data around revisional surgeries. In some cases, such as within the reconstructive cohort, PROMs completed by different groups of patients after revision appeared to show better outcomes than those completed before revision. Although this comparison involves different patients (rather than prerevision and postrevision scores from the same individuals), it raises the possibility that revisional surgery, particularly in the context of complications, may lead to improved patient satisfaction or experience. However, the number of patients who completed PROMs both before and after a revision was very limited. Even without this additional constraint, the volume of cases captured under “revision due to complication” in Tables 1 and 2 is already relatively small, limiting the ability to draw reliable conclusions about changes in PROMs over time.

The collection of PROMs is resource intensive and provides a challenge for clinicians and the registry. Since PROM commencement in 2017, over 65,992 participants were contacted to complete the BREAST-Q IS. We were able to demonstrate the predictive value of the BREAST-Q IS and established that 3 of its questions had a significant association with revision due to complications excluding patient preference within 2 years of the first completed PROM response. With the statistical methods utilized, we found that PROMs have useful predictive properties for breast device registries, however findings should be extrapolated with caution. Despite the aforementioned limitations, our findings support the feasibility of employing registry-based PROMs to predict revisional surgery and point to some valuable directions for future research: evaluating PROM score trajectories across the surgical pathway, and a more detailed investigation into the predictive utility of the BREAST-Q IS at each specific time point of PROM collection.

Our study has many strengths. We minimized the effects of missing data by filtering out patients who did not complete all 5 PROM questions, a previously validated method for handling missing data, and to eliminate the possibility of different samples with each PROM univariate regression. Our large sample size increases generalizability and reduces the chance of sampling error, allowing us to more closely approximate the effect on the population of breast implant recipients. The presence of statistically significant findings indicates that the study was not underpowered. The large registry size makes it one of the best sources to investigate this research question and future questions related to PROMs and surgery outcomes.

## CONCLUSIONS

In conclusion, revisions following breast implant surgery due to complications pose a burden on both patients and surgeons. In this study we investigated the ability of the PROM, BREAST-Q IS, to predict revisional surgery due to complications within 2 years of the first completed PROM response among women from the ABDR. We found that 3 BREAST-Q IS questions, feel, rippling, and tightness for the reconstructive cohort, and 3 BREAST-Q IS questions for the cosmetic cohort, look, rippling, and tightness, were able to predict revisional surgery due to specific complications, excluding patient preference, for both breast reconstructive patients and cosmetic patients. PROMs can be employed to assist with diagnosis, revisions, and complications in device surgery. The statistical models in this study provide a template for future studies in establishing the predicative ability of PROMs.

## Supplemental Material

This article contains supplemental material located online at https://doi.org/10.1093/asj/sjaf128.

## Supplementary Material

sjaf128_Supplementary_Data
