# Supplementary material for: The BREAST-Q Implant Surveillance Module (BREAST-Q IS) As a Predictor of Breast Implant Revisional Surgery
Source: Aesthet Surg J. 2025 Jun 28;45(12):1241–51. doi: 10.1093/asj/sjaf128 (PMC12620023; doi:10.1093/asj/sjaf128)
Supplement: sjaf128_Supplementary_Data [file sjaf128_supplementary_data.zip › SUPPLEMENTAL_Figure_1.docx]

**Supplemental Figure 1.** Area Under ROC Curve for Final Multivariate Model: Reconstruction Cohort

**
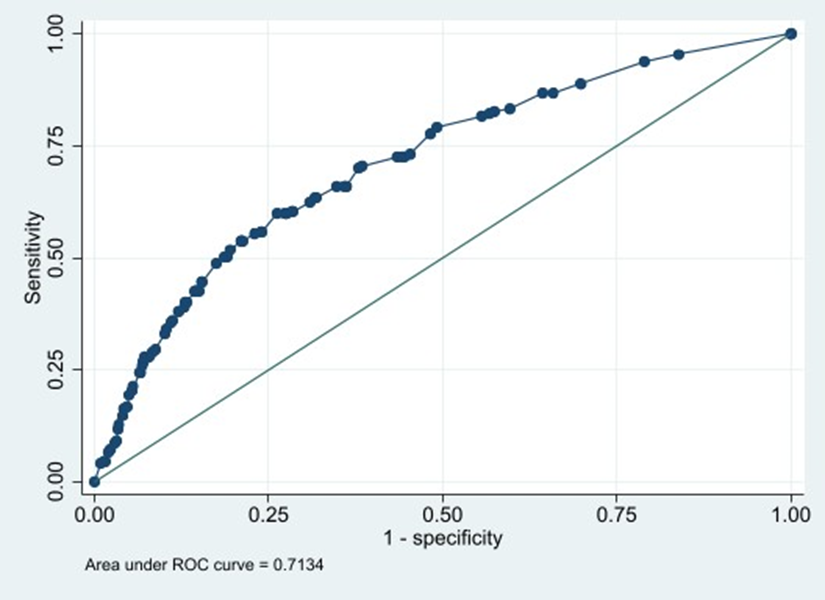
**

AUC = 0.7134
