# Supplementary material for: The BREAST-Q Implant Surveillance Module (BREAST-Q IS) As a Predictor of Breast Implant Revisional Surgery
Source: Aesthet Surg J. 2025 Jun 28;45(12):1241–51. doi: 10.1093/asj/sjaf128 (PMC12620023; doi:10.1093/asj/sjaf128)
Supplement: sjaf128_Supplementary_Data [file sjaf128_supplementary_data.zip › SUPPLEMENTAL_Table_1.docx]

**Supplemental Table 1.** Univariate Model for the BREAST-Q IS Individual Questions at 2-Year Follow-up for Reconstructive and Cosmetic Implants for Specific Complications

RECONSTRUCTION: 197 breast level revisions with complication within 2 years

Device malposition (94 revisions with this issue)

|  | OR (95% CI) | p-value |
| --- | --- | --- |
| PROMs look | 0.43 (0.33-0.55) | <0.001 |
| PROMs feel | 0.42 (0.32-0.54) | <0.001 |
| PROMs rippling | 0.44 (0.34-0.56) | <0.001 |
| PROMs pain | 0.58 (0.46-0.72) | <0.001 |
| PROMs tightness | 0.64 (0.54-0.76) | <0.001 |

Capsular contracture (97 revisions with this issue)

|  | OR (95% CI) | p-value |
| --- | --- | --- |
| PROMs look | 0.68 (0.53-0.86) | 0.002 |
| PROMs feel | 0.58 (0.46-0.74) | <0.001 |
| PROMs rippling | 0.74 (0.58-0.93) | 0.011 |
| PROMs pain | 0.80 (0.62-1.03) | 0.081 |
| PROMs tightness | 0.66 (0.56-0.77) | <0.001 |

Device rupture / deflation (14 revisions with this issue)

|  | OR (95% CI) | p-value |
| --- | --- | --- |
| PROMs look | 0.55 (0.36-0.85) | 0.007 |
| PROMs feel | 0.54 (0.29-1.00) | 0.051 |
| PROMs rippling | 0.64 (0.37-1.09) | 0.100 |
| PROMs pain | 0.79 (0.40-1.53) | 0.479 |
| PROMs tightness | 1.34 (0.78-2.30) | 0.287 |

Skin scarring (19 revisions with this issue)

|  | OR (95% CI) | p-value |
| --- | --- | --- |
| PROMs look | 0.52 (0.30-0.90) | 0.020 |
| PROMs feel | 0.46 (0.28-0.76) | 0.002 |
| PROMs rippling | 0.72 (0.40-1.30) | 0.271 |
| PROMs pain | 0.68 (0.38-1.21) | 0.187 |
| PROMs tightness | 0.48 (0.34-0.69) | <0.001 |

Seroma / haematoma (6 revisions with this issue)

|  | OR (95% CI) | p-value |
| --- | --- | --- |
| PROMs look | 1.69 (0.51-5.61) | 0.387 |
| PROMs feel | 0.96 (0.93-1.01) | 0.086 |
| PROMs rippling | 0.84 (0.18-3.92) | 0.820 |
| PROMs pain | 0.34 (0.12-0.94) | 0.039 |
| PROMs tightness | 0.43 (0.21-0.91) | 0.026 |

Deep wound infection (8 revisions with this issue)

|  | OR (95% CI) | p-value |
| --- | --- | --- |
| PROMs look | 0.60 (0.18-2.02) | 0.405 |
| PROMs feel | 0.41 (0.12-1.37) | 0.148 |
| PROMs rippling | 0.42 (0.13-1.30) | 0.133 |
| PROMs pain | 0.57 (0.24-1.39) | 0.217 |
| PROMs tightness | 0.79 (0.34-1.81) | 0.575 |

## COSMETIC: 249 breast level revisions with complication within 2 years

Device malposition (107 revisions with this issue)

|  | OR (95% CI) | p-value |
| --- | --- | --- |
| PROMs look | 0.36 (0.29-0.45) | <0.001 |
| PROMs feel | 0.48 (0.37-0.62) | <0.001 |
| PROMs rippling | 0.54 (0.42-0.69) | <0.001 |
| PROMs pain | 0.62 (0.49-0.80) | <0.001 |
| PROMs tightness | 0.61 (0.48-0.77) | <0.001 |

Capsular contracture (115 revisions with this issue)

|  | OR (95% CI) | p-value |
| --- | --- | --- |
| PROMs look | 0.42 (0.33-0.53) | <0.001 |
| PROMs feel | 0.48 (0.37-0.61) | <0.001 |
| PROMs rippling | 0.53 (0.42-0.68) | <0.001 |
| PROMs pain | 0.63 (0.50-0.79) | <0.001 |
| PROMs tightness | 0.56 (0.45-0.69) | <0.001 |

Device rupture / deflation (37 revisions with this issue)

|  | OR (95% CI) | p-value |
| --- | --- | --- |
| PROMs look | 0.76 (0.48-1.20) | 0.237 |
| PROMs feel | 0.82 (0.48-1.38) | 0.446 |
| PROMs rippling | 0.59 (0.41-0.84) | 0.004 |
| PROMs pain | 0.71 (0.51-0.99) | 0.045 |
| PROMs tightness | 0.60 (0.43-0.85) | 0.003 |

Skin scaring (19 revisions with this issue)

|  | OR (95% CI) | p-value |
| --- | --- | --- |
| PROMs look | 0.30 (0.20-0.47) | <0.001 |
| PROMs feel | 0.47 (0.28-0.79) | 0.005 |
| PROMs rippling | 0.43 (0.24-0.78) | 0.005 |
| PROMs pain | 0.45 (0.31-0.65) | <0.001 |
| PROMs tightness | 0.55 (0.37-0.82) | 0.003 |

Seroma / haematoma (4 revisions with this issue)

|  | OR (95% CI) | p-value |
| --- | --- | --- |
| PROMs look | 0.34 (0.16-0.74) | 0.007 |
| PROMs feel | 0.62 (0.18-2.20) | 0.462 |
| PROMs rippling | 0.45 (0.15-1.38) | 0.163 |
| PROMs pain | 0.76 (0.46-1.25) | 0.280 |
| PROMs tightness | 0.34 (0.22-0.52) | <0.001 |

Deep wound infection (1 revision with this issue)

|  | OR (95% CI) | p-value |
| --- | --- | --- |
| PROMs look | 0.54 (0.52-0.55) | <0.001 |
| PROMs feel | 0.47 (0.45-0.48) | <0.001 |
| PROMs rippling | 0.45 (0.44-0.46) | <0.001 |
| PROMs pain | 0.60 (0.59-0.62) | <0.001 |
| PROMs tightness | 0.30 (0.29-0.32) | <0.001 |
