# Supplementary material for: The BREAST-Q Implant Surveillance Module (BREAST-Q IS) As a Predictor of Breast Implant Revisional Surgery
Source: Aesthet Surg J. 2025 Jun 28;45(12):1241–51. doi: 10.1093/asj/sjaf128 (PMC12620023; doi:10.1093/asj/sjaf128)
Supplement: sjaf128_Supplementary_Data [file sjaf128_supplementary_data.zip › SUPPLEMENTAL_Table_2.docx]

**Supplemental Table 2.** Univariate Area Under ROC Curve for Each PROM Question and Variable Type for Revision Due to Complication: Reconstructive

| PROM | Categorical variable  AUC | Continuous Linear  AUC | Continuous Quadratic  AUC  (p-value of quadratic term) |
| --- | --- | --- | --- |
| Look | 0.6599 | 0.6599 | 0.6599  (0.870) |
| Feel | 0.6666 | 0.6666 | 0.6666  (0.604) |
| Rippling | 0.6607 | 0.6508 | 0.6508  (0.277) |
| Pain | 0.6152 | 0.6152 | 0.6152  (0.074) |
| Tightness | 0.6451 | 0.6451 | 0.6451  (0.696) |
