# Supplementary material for: The BREAST-Q Implant Surveillance Module (BREAST-Q IS) As a Predictor of Breast Implant Revisional Surgery
Source: Aesthet Surg J. 2025 Jun 28;45(12):1241–51. doi: 10.1093/asj/sjaf128 (PMC12620023; doi:10.1093/asj/sjaf128)
Supplement: sjaf128_Supplementary_Data [file sjaf128_supplementary_data.zip › SUPPLEMENTAL_Table_3.docx]

**Supplemental Table 3.** Univariate Area Under ROC Curve for Each PROM Question and Variable Type for Revision Due to Complication: Cosmetic

| PROM | Categorical variable  AUC | Continuous Linear  AUC | Continuous Quadratic  AUC  (p-value of quadratic term) |
| --- | --- | --- | --- |
| Look | 0.6918 | 0.6918 | 0.6918  (0.718) |
| Feel | 0.6520 | 0.6520 | 0.6520  (0.495) |
| Rippling | 0.6204 | 0.6204 | 0.6204  (0.470) |
| Pain | 0.6248 | 0.6231 | 0.6231  (0.605) |
| Tightness | 0.5966 | 0.5959 | 0.5959  (0.881) |
